# The Hypoactivity Associated with the Repeated Exposure to Atrazine Is Related to Decreases in the Specific Binding to D1-DA Receptors in the Striatum of Rats

**DOI:** 10.1155/2017/2169212

**Published:** 2017-12-06

**Authors:** José Abraham Márquez-Ramos, Isela Hernández-Plata, Mauricio Díaz-Muñoz, Verónica M. Rodríguez

**Affiliations:** ^1^Departamento de Neurobiología Conductual y Cognitiva, Instituto de Neurobiología, Universidad Nacional Autónoma de México, Boulevard Juriquilla 3001, 76230 Querétaro, QRO, Mexico; ^2^Departamento de Neurobiología Molecular y Celular, Instituto de Neurobiología, Universidad Nacional Autónoma de México, Boulevard Juriquilla 3001, 76230 Querétaro, QRO, Mexico

## Abstract

The herbicide atrazine (ATR) has a potential toxic effect on the neuronal circuits of the brain, specifically on two major dopaminergic pathways: the nigrostriatal and mesolimbic circuits. In this work, we repeatedly exposed adult male Sprague-Dawley rats to 6 injections of 100 mg ATR/kg of body weight (for two weeks) and one saline injection two days after ATR administration. Locomotor activity was assessed for 15 minutes and/or 2 hours after ATR or saline injection and 2 months after the final ATR administration. The specific binding of [^3^H]-SCH23390 to D1-DA receptors and that of [^3^H]-Spiperone to D2-DA receptors in the dorsal and ventral striatum were assessed 2 days and 2 months after ATR treatment. ATR administration resulted in immediate, short- and long-term hypoactivity and reduced specific binding of [^3^H]-SCH23390 in the dorsal striatum of rats evaluated 2 months after the last ATR injection. The specific binding of [^3^H]-SCH23390 in the ventral striatum and the specific binding of [^3^H]-Spiperone in the dorsal and ventral striatum remained unchanged at 2 days or 2 months after ATR treatment. These results, together with previous findings of our group, indicate that the nigrostriatal system is a preferential target for ATR exposure.

## 1. Introduction

The use of artificial chemical compounds in the environment has dramatically increased worldwide over the last few decades. Pesticides used in agriculture are among the most toxic substances because of their high chemical stability, resistance to metabolism, and ease of incorporation into cellular environments due to their lipophilic properties [1, 2]. The herbicide atrazine (ATR, 2-chloro-4-ethylamino-6-isopropylamino-2,4,6-triazine), a member of the chlorotriazine family, was introduced in the 1950s as a broad-spectrum herbicide, and today it is commonly used worldwide. Its mechanism of action is associated with the inhibition of the plastoquinone-binding protein of plants [2], and it is used to control weeds mainly found in corn crops but also in sorghum, sugar cane, and other crops [3]. Due to its wide usage, ATR is a ubiquitous water contaminant [3]. It is almost nonvolatile and its half-life in neutral condition is about 200 days but ranges from 4 to 57 weeks depending on various environmental factors such as pH, moisture content, temperature, and microbial activity [4].

Several studies point to the deleterious effects of ATR on the nigrostriatal and mesolimbic dopaminergic systems [5–7]. The nigrostriatal system, which is strongly related to motor function, originates in the zona compacta of the substantia nigra (SNpc) and sends projections to the dorsal striatum (STR). The mesolimbic dopamine system, on the other hand, originates from the dopaminergic cells in the ventral tegmental area (VTA) and projects to the ventral striatum (nucleus accumbens; NAcc) [8], which is important for motivational functions including reward processing and reinforcement learning [9, 10]. Studies have shown that these dopaminergic pathways are damaged by ATR exposure, because changes have been detected at molecular, cellular, and behavioral levels [5–7, 11, 12]. In this context, it has been shown that ATR exposure alters brain dopamine (DA) and serotonin (5-HT) homeostasis, suggesting that ATR modifies tyrosine and tryptophan metabolism. Additionally, several studies found that ATR exposure decreased striatal DA levels [5–7], an effect that is associated with the loss of tyrosine hydroxylase (TH+) positive dopaminergic neurons in the SNpc [13] in rats and in the SNpc and VTA in mice [5]. The negative effects of ATR on dopaminergic metabolism were demonstrated by using rat striatal vesicles and synaptosomes [14], which were exposed to a concentration range of ATR (0.1–250 *μ*M) for 15 min. A dose-dependent decrease in vesicular DA uptake was observed, being the reduction significant at 1 *μ*M ATR. ATR also increased the ratio of synaptosomal/vesicular (DAT/VMAT-2) uptake in a dose-dependent manner.

Exposure to ATR causes immediate [15] and short-term hypoactivity. Such hypoactivity did not occur when the 24 h cycle was evaluated 10 days after completing ATR treatment, but rats exhibited hyperactivity when injected with amphetamine sulfate 2 months after ATR treatment, revealing hidden effects of ATR on dopaminergic systems [7].

Although ATR modifies dopaminergic neurotransmission at several levels, the effects of ATR exposure on DA receptors have been scarcely studied. The D1- and D2-like dopaminergic receptors are G protein-coupled receptors located throughout the brain, including the STR, NAcc, and olfactory tubercle. Several studies have shown the role of these receptors in motor control, reward and reinforcement, and learning and memory [16, 17]. Previous findings have demonstrated that mRNA expression in D1-DA, D2-DA, D1-DA1a, and D4-DA receptors remained unchanged in the STR, NAcc, or substantia nigra of rodents exposed to ATR [7, 11]. However, other pesticides, such as 2,4-D, parathion and glyphosate, could modify other receptor characteristics, for instance, affinity, density, or membrane translocation [18–20].

The present project was aimed at analyzing alternative ATR dopaminergic targets by exploring the binding properties of D1- and D2-dopaminergic receptors in the nigrostriatal (striatum) and mesolimbic system (nucleus accumbens) at two ATR postexposure intervals in the rat.

## 2. Materials and Methods

### 2.1. Subjects

Thirty-four male Sprague-Dawley rats (250–300 g) were obtained from Harlan Laboratories Inc. (Mexico City, Mexico). Rats were kept under a 12 h inverted light/dark cycle (lights on at 20:00 h) with access to food and water ad libitum. Animals were habituated to vivarium conditions for one week before starting the experiments. Experiments were approved by the local Committee of Bioethics and carried out according to the Official Mexican Standard NOM-062-ZOO-1999, which complies with the guidelines of the Institutional Animal Care and Use Committee Guidebook (NIH Publication 80-23, Bethesda, MD, USA, 1996).

### 2.2. Chemicals

ATR was purchased from Chem Service (West Chester, PA, USA). Reagents for western blotting were obtained from BioRad (Hercules, CA, USA). [^3^H]-SCH23390 (dopamine D1-like receptor antagonist) and [^3^H]-Spiperone (dopamine D2-like receptor antagonist) were purchased from Perkin-Elmer (Boston, MA, USA). All other reagents were obtained from Sigma-Aldrich (St. Louis, MO, USA), unless otherwise stated.

### 2.3. Experimental Design

Rats were divided at random into two groups. One group received 6 intraperitoneal (IP) injections of 1% methylcellulose (1% MC, vehicle), while the other received 6 IP injections of 100 mg ATR/kg body weight (BW) over a two-week period (3 injections per week at 48-h intervals). Forty-eight hours after the last ATR or vehicle administration, rats received an IP saline injection. ATR was administered in accordance with “Health Effects Test Guidelines OPPTS 870.6200 Neurotoxicity Screening,” available at the website (https://www.regulations.gov/#!documentDetail;D=EPA-HQOPPT-2009-0156-0041). Locomotor activity was evaluated for 15 min before and for 2 h immediately after each injection of vehicle, ATR, or saline. Rats were divided into two groups and euthanatized by decapitation. One group of rats was sacrificed 2 days after the last ATR injection, while the remaining rats were sacrificed 2 months after ATR treatment. Their brains were dissected on ice. Both the whole dorsal STR and ventral striatum (NAcc) were isolated and stored at −80°C for posterior analysis. Ten animals were evaluated for locomotor activity only on one occasion, 2 months after ATR treatment or saline (for 15 min).

### 2.4. Effects of ATR Exposure on Locomotor Activity

To assess the effects of ATR exposure on locomotor activity, we used a previously reported protocol with an automatic recording system (Digiscan Animal Activity Monitors—Accuscan Inc., Columbus, OH, USA) [7]. Briefly, animals were placed in acrylic chambers (40 cm × 40 cm × 40 cm) surrounded by horizontal and vertical infrared beams. Locomotor activity was recorded for 15 min before (exploratory activity) and for 2 h immediately after ATR administration (100 mg ATR/kg BW) or 1% MC. Two days after the last ATR or MC injection rats received an IP saline injection, and locomotor activity was recorded with the same protocol. Some rats only underwent a 15-min evaluation 2 months after ATR treatment. The parameters used to evaluate the effects of ATR on locomotor behavior were total distance (expressed as the distance traveled in centimeters by the rat in a specific period), vertical activity (described as total number of beam interruptions of the vertical sensor during a given period), and stereotypy counts (defined as the number of beam breaks due to repetitive movements that occurred during a determined period).

### 2.5. Radiobinding Assay for D1- and D2-DA Receptors

STR or NAcc from both hemispheres of each animal (*n* = 6 per group) was homogenized in ice-cold 50 mM Tris-HCl buffer (1 : 20 w/v, pH 7.4) containing 1% protease and phosphatase cocktail inhibitors (Sigma-Aldrich). Subsequently, each homogenized sample was divided into two, and each half was used to assess either D1-like or D2-like DA receptors as previously reported by our group [20].

### 2.6. Statistical Analysis

Spontaneous locomotor activity and body weight were analyzed using two-way repeated-measures ANOVA followed by post hoc tests (Student's *t*-test) for significant main effects or interactions. The binding assay was analyzed with an unpaired *t*-test. Data analyses were carried out using StatView version 5.0 (SAS Institute Inc., Cary, NC, USA). Statistical significance was defined as *p* < 0.05.

## 3. Results

### 3.1. Body Weight and General Appearance

After 2 weeks of treatment, ATR exposure did not cause alterations in the body weight or general appearance of rats exposed to 100 mg ATR/kg BW in comparison to the control. Regarding body weight, no effects of ATR treatment [*F*(1,32) = 2.55, *p* = 0.1201] or interaction (treatment by injection number) [*F*(6,192) = 1.450, *p* = 0.1977] were found, but significant effects of injection number (*F*(6,192) = 126.81, *p* < 0.0001) were found, with rats showing a decrease in body weight along injections of vehicle or ATR as shown in Table 1.

### 3.2. Effects of Atrazine Exposure on Locomotor Activity

#### 3.2.1. ATR Exposure Decreased Exploratory Behavior

**Figure 1:**
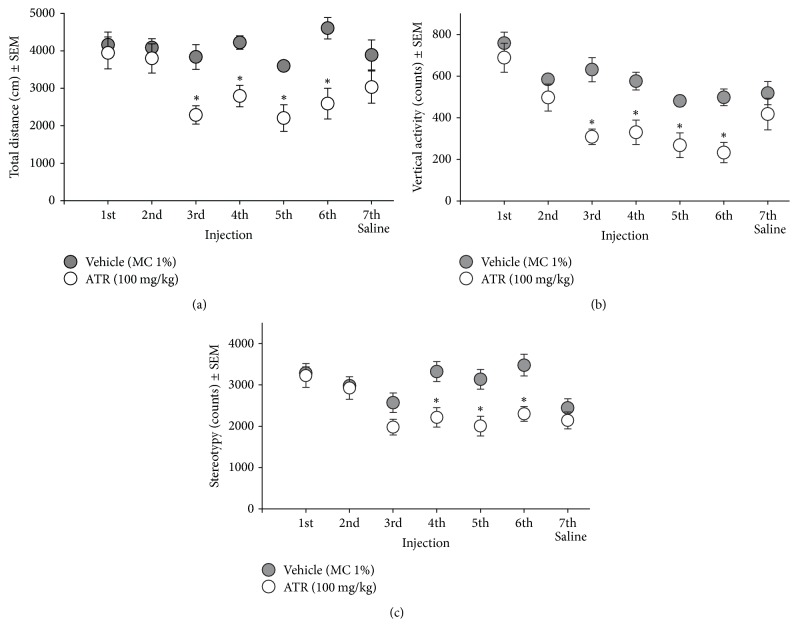
Exploratory locomotor activity [(a) total distance, (b) vertical activity, and (c) stereotypy] recorded 15 min immediately before the IP administration of 100 mg ATR/kg BW (six injections over a two-week period) and one saline IP injection (48 h after the last ATR injection). With the exception of the first injection, these exploratory recordings occurred in intervals of 48 h after the preceding injection. ^*∗*^Different from vehicle, *p* < 0.05. *n* = 10.

During the exploration of locomotor chambers (15 min before injection of vehicle or ATR), the ATR group differed from control group in total distance [*t*'s (18) = (−3.32)–(−5.10), *p* = 0.002–0.0001] and vertical activity [*t*'s (18) = (−3.53)–(−4.69), *p* = 0.001–0.0001] at the third ATR injection. Decreases in stereotypy were observed at the fourth ATR injection [*t*'s (18) = (−3.88)–(−5.36), *p* = 0.0004–0.0001] (Figure 1).

#### 3.2.2. Atrazine Exposure Decreased Spontaneous Locomotor Activity

**Figure 2:**
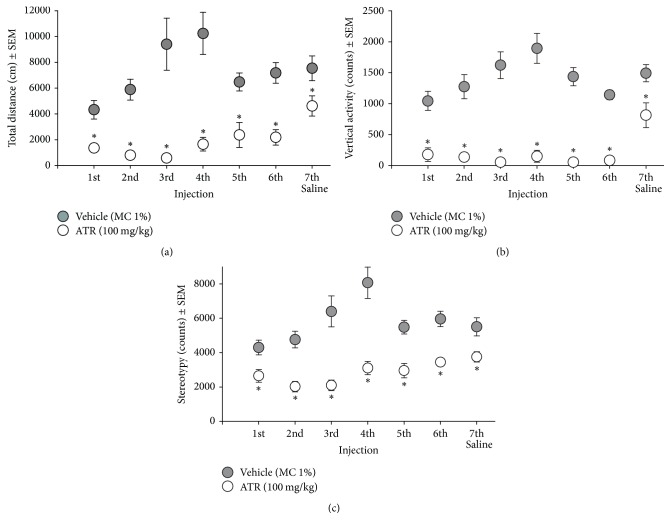
Locomotor activity [(a) total distance, (b) vertical activity, and (c) stereotypy] recorded for 2 h immediately after the IP administration of 100 mg ATR/kg BW (six injections over a two-week period) and one saline IP injection (48 h after the last ATR injection). These recordings occurred in intervals of 48 h after the preceding injection. ^*∗*^Different from vehicle, *p* < 0.05. *n* = 10.

ATR administration caused significant hypoactivity immediately after injection (Figure 2). All ATR injections showed statistically significant decrements in comparison to the vehicle group in total distance [*t*'s (18) = (−2.82)–(−6.79), *p* = 0.007–0.0005], vertical activity [*t*'s (18) = (−4.40)–(−7.68), *p* ≤ 0.0001], and stereotypy [*t*'s (18) = (−2.07)–(−5.70), *p* = 0.04–0.0001]. Hypoactivity in the ATR-treated group lasted until rats received a saline injection (total distance, *t* (18) = −2.14, *p* = 0.04; vertical activity, *t* (18) = −2.35, *p* = 0.02; and stereotypy, *t* (18) = −2.44, *p* = 0.02).

#### 3.2.3. Atrazine Exposure Decreased Locomotor Activity 2 Months after ATR Treatment

**Figure 3:**
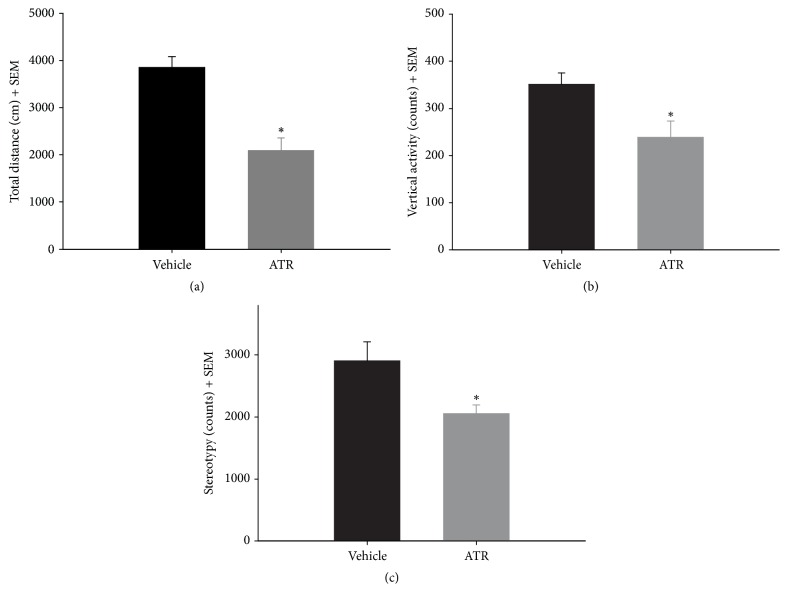
Exploratory locomotor activity [(a) total distance, (b) vertical activity, and (c) stereotypy] recorded for 15 min 2 months after the last ATR injection. ^*∗*^Different from vehicle, *p* < 0.05. *n* = 5.

Rats were hypoactive 2 months after the last ATR injection. Hypoactivity in the ATR-treated group was identified in total distance, *t* (8) = −5.051, *p* = 0.0010; vertical activity, *t* (8) = −2.652, *p* = 0.0292; and stereotypy, *t* (8) = −2.493, *p* = 0.0374 (Figure 3).

### 3.3. Atrazine Exposure Decreased Specific Binding of the Antagonist [^3^H]-SCH23390 to D1-DA Receptors in STR 2 Months after ATR Treatment

**Figure 4:**
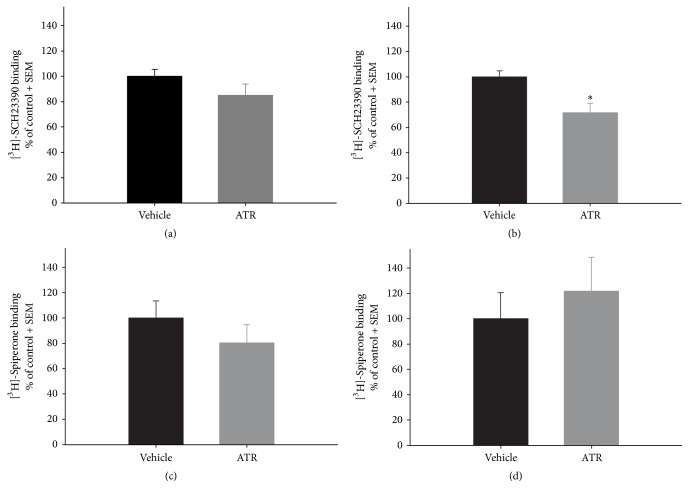
Specific binding of the antagonists [^3^H]-SCH23390 ((a) and (b)) and [^3^H]-Spiperone ((c) and (d)) to D1- and D2-DA receptors, respectively, in the striatum of rats repeatedly exposed to 100 mg ATR/kg, measured 2 days ((a) and (c)) or 2 months ((b) and (d)) after the last ATR injection. Absolute values (nmol/mg protein ± SE) for vehicle groups: (a) 305.47 ± 16.87, (b) 116.93 ± 5.41, (c) 55.90 ± 7.6, and (d) 12.66 ± 2.61; *n* = 5-6. ^*∗*^Different from vehicle, *p* < 0.05.

**Figure 5:**
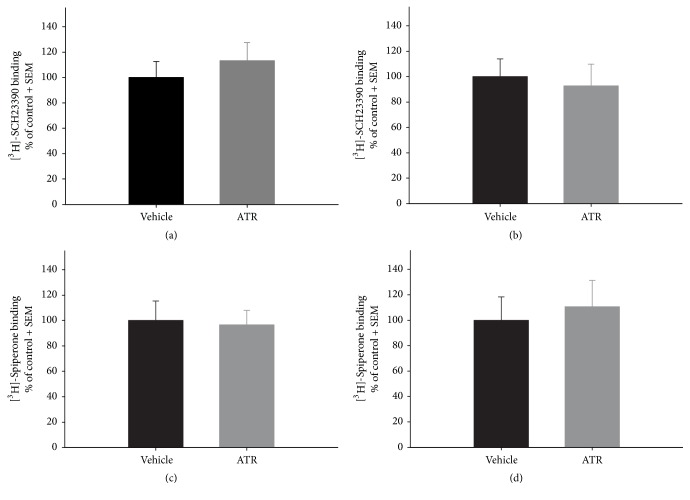
Specific binding of the antagonists [^3^H]-SCH23390 ((a) and (b)) and [^3^H]-Spiperone ((c) and (d)) to D1- and D2-DA receptors, respectively, in the nucleus accumbens of rats repeatedly exposed to 100 mg ATR/kg, measured 2 days ((a) and (c)) or 2 months ((b) and (d)) after the last ATR injection. Absolute values (nmol/mg protein ± SE) for vehicle groups: (a) 161.58 ± 20.61, (b) 44.04 ± 6.21, (c) 33.18 ± 5.12, and (d) 11.63 ± 2.15; *n* = 5-6.

ATR treatment had no effect on STR for the specific binding of [^3^H]-SCH23390 to D1-DA receptors 2 days after ATR treatment (Figure 4(a)). However, a significant decrease was identified in the specific binding of [^3^H]-SCH23390 to D1-DA receptors in the STR of animals treated and sacrificed 2 months after ATR treatment [*t* (10) = −3.205, *p* = 0.0094] in comparison to control animals (Figure 4(b)). Conversely, there were no observed effects on the specific binding of [^3^H]-Spiperone to D2-DA receptors 2 days or 2 months after ATR treatment (Figures 4(c) and 4(d)). Meanwhile, there were no effects observed on the specific binding of [^3^H]-SCH23390 to D1-DA receptors or [^3^H]-Spiperone to D2-DA receptors in the NAcc of tissue collected 2 days or 2 months after ATR treatment (Figure 5).

## 4. Discussion

The present study showed that repeated exposure to 100 mg ATR/kg BW over a 2-week period causes immediate and short- and long-term hypoactivity, as well as a reduction in the specific binding of the antagonist [^3^H]-SCH23390 in the STR collected 2 months after ATR exposure.

Hypoactivity data associated with the ATR treatment presented here agrees with earlier studies in adult rodents [7, 11, 15, 21]. In this respect, acute and repeated ATR administration decreased locomotor activity, which lasted up to 48 h and caused hypoactivity up to 5 days after the last ATR administration [7]. In this study we found that hypoactivity was still present 2 months after ATR exposure. Interestingly, in a previous study, we found that rats presented hyperactivity when they received an IP injection of amphetamine sulfate, a dopamine agonist that increases DA release and reduces its reuptake into the presynaptic terminal [15], revealing a hidden alteration in the dopaminergic system [7]. In an ATR treatment protocol similar to the one used in this study, we found decreases in striatal levels of DA, DOPAC, HVA, and 5-HIAA five days after ending ATR treatment [7].* In vivo* studies showed that multiple exposure scenarios (i.e., acute, repeated, short-term, or chronic exposures and perinatal exposure to ATR) disrupt striatal DA homeostasis [5–7, 11–14].* In vitro* studies using PC12 cells incubated with ATR for up to 24 h showed that the herbicide treatment produced a dose-dependent reduction in intracellular DA [22]. In addition, another study using the human dopaminergic neuroblastoma cell line SH-SY5Y reported that ATR causes diminution in cell proliferation [23]. Taken together, these studies have demonstrated dopaminergic susceptibility to ATR both* in vivo* and* in vitro*.

An important contribution of this paper is the evaluation of the effects of ATR exposure on DA receptor binding. Our group reported that expression of D1-DA and D2-DA receptor mRNA in STR or NAcc was not modified by ATR exposure (100 mg ATR/kg, 6 injections a week for 2 weeks) when rats were sacrificed 2 days or 3 months after ending ATR treatment [7]. However, our data in Figure 4 show that ATR could alter other characteristics of D1-DA receptors. Particularly, we found that the specific binding to D1-DA receptors decreased in STR, which was accompanied by hypoactivity in rats 2 months after ATR treatment. This finding is supported by reports on other agrochemicals, such as 2-4 D, parathion, and glyphosate, since they can modify the affinity, density, and/or translocation of the D1 or D2 receptors in the brain [18–20]. As shown in Figure 4(b), ATR affected striatal D1-DA receptors but not striatal D2-DA receptors or accumbal D1-DA or D2-DA receptors.

In this study, ATR exposure caused hypoactivity all through the experiment, and this hypoactivity was still present two months after ending ATR treatment. Additionally, ATR exposure reduced the specific binding of D1 DA receptors, 2 months after ending ATR exposure, but not at 48 h of exposure. Both findings may be the result of the effect that ATR causes on dopamine metabolism, such as decreases in the content of DA and its metabolites [5, 6, 11, 13, 15]. While the early decrease in locomotor activity may be due to a pharmacological-type effect of ATR and the long-term effects on activity, as well as the decrease in specific binding which may be the result of cell loss or plastic changes induced by the pesticide. The lack of binding changes during early exposure observed in this study is similar to what Lin et al. [11] and Rodriguez et al. [15] observed. These authors did not find changes in the messenger expression of D1 or D2 type DA receptors when evaluated after 10 days of ATR treatment or 2 days after ending ATR treatment.

The reduced specific binding of [^3^H]-SCH23390 in STR can be interpreted as a diminution in the availability of D1-DA receptors that could be related to the hypoactivity caused by ATR exposure 2 months after ATR treatment. In this regard, the relationship between D1-DA receptors and motor control has been thoroughly studied [24–28]. Acute or chronic administration of the dopamine D1-like antagonist (SCH23390) caused catalepsy in rats [29, 30], while administration of dopamine D1-like agonists (SKF) caused stimulation of locomotor activity [31].

In general, ATR herbicide causes presynaptic changes since the effects reported are mainly on striatal DA synthesis, reuptake, or storage. However, our results about the effects associated with striatal D1-DA receptors indicate that ATR may also cause postsynaptic alterations, since striatal D1-DA receptors are localized primarily in the postsynaptic striatal GABAergic neurons [32], suggesting that GABAergic neurotransmission could be affected.

In the present manuscript, the vulnerability of the DAergic pathways upon agrochemical exposure is evident. In this respect, we found a reduction of D1-DA receptors in STR but not in NAcc after ATR treatment. Some agrochemicals that have different mechanisms of toxicity than those of ATR, such as paraquat and glyphosate, are reported to cause alterations in dopaminergic pathways. Paraquat exposure affects the nigrostriatal pathway, producing alterations in dopaminergic markers [24, 33, 34]. In contrast, the herbicide glyphosate modified the specific binding of [^3^H]-SCH2339 in NAcc, but not in STR [20]. These actions could be explained by morphological and physiological differences between the nigrostriatal or mesolimbic pathways such as neuron soma size, neuron density, DAT expression, and synaptic transmission [25–28, 35].

In conclusion, this study showed that repeated exposure to the herbicide ATR induced hypoactivity in rats, which could be associated with reduced levels of D1-DA receptors in the STR. These results further support the potential toxic effects of ATR on the nigrostriatal dopaminergic system.

## Figures and Tables

**Table 1:** Rats body weight before, during, and after ATR exposure (percent of initial weight).

	Vehicle	ATR 100 mg/kg
Injection 1	100 (17)	100 (17)
Injection 2	96.77 ± 0.23 (17)	96.32 ± 0.38 (17)
Injection 3	91.58 ± 0.41 (17)	92.65 ± 0.74 (17)
Injection 4	89.42 ± 0.61 (17)	90.54 ± 0.63 (17)
Injection 5	90.22 ± 0.55 (17)	91.71 ± 0.54 (17)
Injection 6	90.41 ± 0.81 (17)	92.01 ± 0.73 (17)
Injection saline	89.32 ± 0.85 (17)	90.87 ± 0.93 (17)
2 months after ATR	116.65 ± 0.72 (5)	116.53 ± 2.18 (5)

Values are mean ± SEM of percent of basal weight (before ATR or vehicle treatment); mean initial weight values (g) for vehicle (*n* = 17) and ATR (*n* = 17), respectively, were 317.54 ± 4.61 and 295.42 ± 3.33; mean initial weight values (g) for vehicle (*n* = 5) and ATR (*n* = 5), respectively, were 314 ± 10.26 and 296.10 ± 5.65.
